# Early repolarization pattern in adult females with eating disorders

**DOI:** 10.1111/anec.12865

**Published:** 2021-06-11

**Authors:** Tanja Charlotte Frederiksen, Morten Krogh Christiansen, Loa Clausen, Henrik Kjærulf Jensen

**Affiliations:** ^1^ Department of Cardiology Aarhus University Hospital Aarhus Denmark; ^2^ Department of Clinical Medicine Aarhus University, Health Aarhus Denmark; ^3^ Research Unit Department of Child‐ and Adolescent Psychiatry Aarhus University Hospital Aarhus Denmark

**Keywords:** early repolarization pattern, eating disorders, electrocardiogram (ECG)

## Abstract

**Introduction:**

The risk of cardiovascular death is increased in patients with eating disorders (ED), but the background for this is unknown. Early repolarization pattern (ERP) on the electrocardiogram (ECG) has been associated with increased risk of sudden cardiac death.

**Methods:**

We investigated the prevalence of ERP in 233 female patients with anorexia nervosa (AN) and bulimia nervosa (BN) (age 18–35 years) compared with 123 healthy female controls.

**Results:**

Early repolarization pattern was present in 52 (22%) of ED patients (16 (15%) AN patients and 36 (29%) BN patients) and 17 (14%) of healthy controls. When adjusting for age, BMI, heart rate, use of selective serotonin reuptake inhibitors (SSRI), and potassium level, the odds ratio (OR) for ERP was 2.1 (95% CI 1.1–4.2, *p *= .03). There was an increased prevalence of inferolateral ERP in patients with ED compared with healthy controls (OR = 4.3, 95% CI 1.7–11.3, *p *= .003) as well as ERP with a downward/horizontal sloping ST segment (OR = 3.1, 95% CI 1.3–7.6, *p *= .01). Additionally, J‐point elevation >0.2 mV was more prevalent in patients with ED (OR = 3.3, 95% CI 1.1–9.7, *p *= .03).

**Conclusion:**

The prevalence of ERP was increased in patients with ED compared with healthy controls. This finding may provide a possible explanation for the increased cardiovascular mortality in ED patients.

## 1 INTRODUCTION

Patients with anorexia nervosa (AN) and bulimia nervosa (BN) have an increased mortality compared with healthy adults (Chesney et al., 2014), including an increased risk of cardiovascular death (Papadopoulos et al., 2009). Recently, a large register‐based study found that the risk of ventricular tachycardia and syncope is increased in patients with AN compared with the background population (Frederiksen, Krogh Christiansen, et al., 2018), which is in line with the previous theory that an increased risk of QTc prolongation on the ECG, and subsequently polymorphic ventricular tachycardia, could partly explain the increased cardiovascular death in patients with AN (Sachs et al., 2016). However, the three largest studies on the matter found no increased risk of QTc prolongation in patients with AN or BN (Frederiksen, Christiansen, et al., 2018; Frederiksen, Krogh Christiansen, et al., 2018; Krantz et al., 2020). Therefore, the background for the increased risk of cardiovascular death in these patients is still unresolved.

Several studies have investigated the clinical and prognostic value of early repolarization pattern (ERP) on the ECG, which has been found to be associated with an increased risk of ventricular fibrillation (VF) and sudden cardiac death (SCD) (Cheng et al., 2017; Junttila et al., 2014; Pieroni et al., 2008). However, the prevalence of this potentially malignant finding has yet to be investigated in patients with ED. Thus, we set out to study the prevalence of ERP in patients with AN or BN compared with healthy controls.

## 2 METHODS

### 2.1 Study population

The study population comprised 233 patients included from the Centre for Eating Disorders, Aarhus University Hospital in the period 1997–2013. All patients underwent a thorough interview, clinical examination (including a 12‐lead ECG), and blood sampling on their first visit to the center. Inclusion criteria were female sex, age >18 years and <35 years at time of inclusion, and full syndrome AN (including both restrictive type and binge‐eating/purging type), atypical AN, BN, or atypical BN according to the Diagnostic and Statistical Manual of Mental Disorders, Fifth Edition (DSM‐5) (Association AP, 2013). Exclusion criteria were missing ECG and prior or current cardiovascular diseases. At the time of inclusion, no patients had any prior or current cardiovascular diseases. The psychopathology of AN and BN patients was assessed using the Eating Disorder Examination (Fairburn, 2008), a standardized diagnostic instrument for interviewers. We recruited 126 healthy women between 18 and 35 years of age as controls through advertisement at university and student environments. They were all examined at the cardiology outpatient clinic by the same examiner. First, a psychiatric assessment was performed based on the diagnostic criteria for EDs from the DSM‐5. Furthermore, the healthy controls were asked about prior and current somatic diseases, as well as other psychiatric disorders. Blood pressure, ECG, weight, and height were measured, and blood samples were obtained on all controls. Controls with prior or current EDs, as well as prior or current cardiovascular diseases, were excluded. Weight was not used as an eligibility criterion. In total, 123 healthy controls were included in the analyses and 3 were excluded and referred for further examination and counseling, two of the three because of previously unknown hypertrophic cardiomyopathy and one because of eating disorder symptoms resembling atypical bulimia nervosa.

### 2.2 Ethics

The study was approved by the Danish Data Protection Agency (record number 2007‐58‐0010) and the Danish Patient Safety Authority (record number 3‐3013‐1636/1/).

### 2.3 ECG measurements

All ECGs were recorded as standard 12‐lead ECGs at a paper speed of 25 mm/s and a voltage of 10 mm/mV. The ECGs were obtained in supine position after at least 5 min of rest. Analyses of ECG intervals were performed manually using digital calipers (EP Calipers, EP Studios, Inc.). All analyses were performed by a single, experienced observer.

ERP was defined according to a consensus paper from 2015 (Macfarlane et al., 2015) and was characterized by the following elements: a QRS notch which occurs on the final 50% of the downslope of an R wave or QRS slur which is a slowing of the inscription of the waveform occurring in the final 50% of the R wave downslope at the end of the QRS complex which merges with the ST segment. Early repolarization was considered present if all the following 3 criteria were met: (1) end‐QRS notch or slur on the downslope of a prominent R wave, (2) Jp (peak of a notch or onset of a slur) equal or more than 0.1 mV in 2 or more contiguous leads, excluding lead V1‐V3, and (3) QRS duration less than 120 milliseconds (ms). ERP was assessed as a binary outcome. Furthermore, when ERP was present, the morphology of the QRS complex was categorized as either notched, slurred, or both. The ST segment in ECGs with ERP was categorized as either upward sloping or horizontal/descending sloping, and ERP was categorized in lead territories as inferior (II, III, aVF), lateral (I, aVL, V5‐6), or both. In addition, J‐point elevation (Jp) >0.2 mV was assessed as a binary variable.

Other ECG measurements included PR interval (ms), QRS durations (ms), QTc interval (ms, corrected for heart rate using Fridericia's formula), Sokolow–Lyon index (mV, amplitude of S in V1 + R in V5 or V6, whichever was greater). Left ventricular hypertrophy was defined as a Sokolow–Lyon index greater than 3.5 mV. Left bundle branch block was defined as a QRS >120 ms, with a dominant S wave in V1‐2 and a broad monophasic R wave in V5‐6. Right bundle branch block was defined by a QRS complex >120 ms with rSR’ morphology in V1‐2 and a wide slurred S waves in lead V5‐6. Incomplete right bundle branch block was defined by a QRS duration <120 ms and right bundle branch block morphology.

### 2.4 Statistical analysis

For patients and controls, characteristics were compared using the chi‐square test, Student's *t*‐test, or the Wilcoxon–Mann‐Whitney test as appropriate. Normality of variables was checked by QQ plots and histograms. Missing data (potassium in 33 patients and BMI in 2 patients) were considered missing at random, and multiple imputations were made using a linear regression model. A number of 20 datasets were imputed for each missing variable. The odds ratio (OR) of ERP on the ECG was assessed and compared between ED patients and the controls using a logistic regression model. The regression model was adjusted for BMI, age, heart rate, use of selective serotonin reuptake inhibitors (SSRI), and potassium level. When assessing OR for the QRS complex morphology, ERP lead localization, J‐point elevation, and ST‐segment slope, we used a univariate logistic regression model, since there were too few outcomes in each group to adjust for other variables. All statistical analyses were performed using Stata Software version 14.2 (StataCorp, College Station, TX).

## 3 RESULTS

### 3.1 Patient characteristics

Patients with ED were younger, had lower BMI, and had heart rate compared with healthy controls. Both systolic and diastolic blood pressure was lower in patients with ED (Table 1). The prevalence of psychopharmacological treatment was higher in patients with ED. Electrolyte levels were similar between groups, except magnesium which was higher in patients with ED. ECG measures were also similar between ED patients and healthy controls except for mean Sokolow–Lyon index and the prevalence of hypertrophy (Sokolow–Lyon index >35 mm), which was higher in healthy controls. None of the patients or healthy controls had complete bundle branch block, but 9.4% of ED patients and 8.1% of controls had incomplete right bundle branch block.

**TABLE 1:** Patient characteristics

	Eating disorder (*n* = 233)	Controls (*n* = 123)	*p*‐Value
Age (years)	22.4 (20.6–24.7)	25.4 (23.2–27.3)	<.001
BMI (kg/m^2^)	19 (4)	23 (3)	<.001
Heart rate (beats per minute)	60 (13)	67 (13)	<.001
Blood pressure (mmHg)			
Systolic	114 (13)	118 (11)	.004
Diastolic	74(10)	77 (9)	.004
Medication			
SSRI	17 (7.3%)	3 (2.4%)	.058
SNRI	3 (1.3%)	0 (0.0%)	.21
TCA	1 (0.4%)	0 (0.0%)	.47
Antipsychotics	1 (0.4%)	0 (0.0%)	.47
Benzodiazepines	3 (1.3%)	0 (0.0%)	.21
NaSSa	3 (1.3%)	0 (0.0%)	.21
Blood samples			
Potassium (mmol/L)	3.8 (0.2)	3.8 (0.2)	.75
Sodium (mmol/L)	140 (2)	140 (2)	.75
Phosphate (mmol/L)	1.13 (0.27)	1.15 (0.20)	.49
Magnesium (mmol/L)	0.86 (0.81–0.90)	0.82 (0.78–0.85)	<.001
Hemoglobin (mmol/L)	8.3 (7.9–9.0)	8.3 (8.0–8.7)	.43
ECG measures			
PR interval, ms	150 (23)	150 (22)	.87
QRS interval (ms)	91 (12)	91 (9)	.73
QTc interval (ms)	401 (23)	400 (20)	.65
Left bundle branch block	0 (0.0%)	0 (0.0%)	‐
Right bundle branch block	0 (0.0%)	0 (0.0%)	‐
Incomplete right bundle branch block	22 (9.4%)	10 (8.1%)	.68
Sokolow–Lyon index	21 (5)	24 (6)	<.001
Sokolow–Lyon index >35 mm	0 (0.0%)	6 (4.9%)	<.001

Note

Data are presented as mean (SD) or median (IQR) for continuous measures, and *n* (%) for categorical measures.

Abbreviations: BMI, body mass index; NaSSA, noradrenergic and specific serotonergic antidepressants; SNRI, serotonin–norepinephrine reuptake inhibitors; SSRI, selective serotonin reuptake inhibitors; TCA, tricyclic antidepressants.

### 3.2 Early repolarization pattern

Early repolarization on the ECG was present in 52 (22%) of ED patients (Table 2), of which 16 (15%) AN patients and 36 BN patients (29%) had ERP. In contrast, ERP was present in 17 (14%) of healthy controls. Using a logistic regression model adjusted for age, BMI, heart rate, SSRI use, and potassium level, the OR for ERP in ED patients was 2.1 (95% CI 1.1–4.2, *p *= .03) compared with healthy controls.

**TABLE 2:** Early repolarization pattern

	Eating disorder, *n* = 233	Controls, *n* = 123	Odds ratio (95% CI)	*p*‐Value
ERP	52 (22%)	17 (14%)	2.1 (1.1–4.2)^a^	.03
QRS morphology				
Notched	8 (3%)	2 (2%)	2.2 (0.4–10.3)	.34
Slurred	44 (19%)	13 (11%)	2.0 (1.0–3.8)	.05
Notched and slurred	0 (0%)	2 (2%)	‐	‐
ERP lead localization				
Inferior	16 (7%)	9 (7%)	0.9 (0.4–2.2)	.87
Lateral	0 (0%)	3 (2%)	‐	‐
Inferolateral	36 (15%)	5 (4%)	4.3 (1.7–11.3)	.003
ST‐segment slope				
Horizontal/downward	32 (14%)	6 (5%)	3.1 (1.3–7.6)	.01
Upward	20 (8%)	10 (8%)	1.1 (0.5–2.3)	.88
J‐point elevation				
>0.2 mV	23 (10%)	4 (3%)	3.3 (1.1–9.7)	.03

^a^ Logistic regression model adjusted for age, BMI, heart rate, SSRI use, and potassium level.

In both ED patients and healthy controls with ERP, a slurred QRS complex was more prevalent than a notched or notched and slurred QRS complex. Compared with healthy controls, there was an increased prevalence of inferolateral ERP in patients with ED with an OR of 4.3 (95% CI 1.7–11.3, *p *= .003). In addition, the prevalence of ERP with a downward/horizontal sloping ST segment was significantly increased in ED patients compared with healthy controls (OR = 3.1, 95% CI 1.3–7.6, *p *= .01). The prevalence of J‐point elevation >0.2 mV was increased in patients with ED compared with healthy controls (OR = 3.3, 95% CI 1.1–9.7, *p *= .03).

## 4 DISCUSSION

To our knowledge, the present study is the first to explore the prevalence of ERP in patients with ED. We found an increased prevalence of ERP in patients with ED compared with healthy controls, both with J‐point elevation >0.10 mV and with J‐point elevation >0.2 mV. In addition, the prevalence of ERP in the inferolateral leads was increased in ED patients as well as ERP with a horizontal/downward sloping ST segment.

Previous studies have found that the risk of VF and SCD in individuals with ERP is largely dependent on lead localization of the ECG pattern (Pieroni et al., 2008; Tikkanen et al., 2009). A community‐based Finnish study of 10.864 middle‐aged individuals found an increased risk of death of cardiac causes in cases with ERP. The association was significantly stronger in ERP in the inferior leads than in the lateral leads (Tikkanen et al., 2009). ERP in both inferior and lateral leads was not associated with death of cardiac causes. However, the prevalence of ERP in both inferior and lateral leads was very low (0.15%). Another study of 206 individuals resuscitated after cardiac arrest with no other identifiable cause found that ERP was more prevalent in these survivors compared with healthy controls (Pieroni et al., 2008). During follow‐up, patients with ERP had a significantly increased risk of a malignant arrhythmic event. Of the patients with ERP, the majority had ERP in both inferior and lateral leads. Thus, the significance of lead localization in ERP remains somewhat unclear.

In a study performed on the before mentioned 10.864 Finnish middle‐aged individuals, the authors examined the morphology of ERP and found the same risk of cardiac death between those with notched and slurred J‐wave ERP (Junttila et al., 2012). However, ERP with a horizontal/descending sloping ST segment was significantly associated with arrhythmic death compared with ERP with an ascending ST segment, which did not carry any increased risk of SCD. This correlates with two studies finding a higher prevalence of ERP in athletes, where the absolute majority presented with an ascending sloped ST segment. In neither of the studies was ERP associated with a risk of arrhythmia or SCD during follow‐up suggesting the existence of a benign form of ERP associated with extensive physical exercise (Noseworthy et al., 2011; Serra‐Grima et al., 2015).

In addition, several studies showed that a higher degree of J‐point elevation (>0.2 mV) was associated with an increased risk of SCD compared with ERP with J‐point elevation limited to 0.1 mV–0.2 mV (Junttila et al., 2012; Junttila et al., 2014; Tikkanen et al., 2009).

The present study gives no explanation to the higher prevalence of ERP in patients with ED. One theory could be an increased vagal tone in ED patients (Kollai et al., 1994; Murialdo et al., 2007), since ERP has been found to be strongly associated with vagal tone (Junttila et al., 2012). However, further studies are needed in order to uncover a possible association between vagal tone and ERP in ED patients.

Our study has several limitations. First, the sample size was relatively modest giving rise to rather large confidence intervals. Second, we do not know whether the presence of ERP among ED patients is associated with an increased cardiovascular risk or whether this may have clinical implications. Further studies are essential in order to examine whether this ECG pattern might be a marker for cardiovascular death in these patients. In addition, ERP has been associated with structural changes in athletes such as larger atrial volume (Wilhelm et al., 2010), higher left ventricular mass, and thicker left ventricular wall (Reinhard et al., 2019). However, we did not have any echocardiographic data on this cohort which could have been useful in order to examine whether ERP is associated with structural cardiac changes in ED patients.

## 5 CONCLUSION

In conclusion, this is the first study to show that the prevalence of ERP is increased in patients with ED compared to healthy controls. Both ERP with J‐point elevation >0.1 and >0.2 mV was more prevalent among patients with ED. The prevalence of inferolateral ERP was increased as well as ERP with a horizontal/downward sloping ST segment. Future studies are needed to assess the association of ERP with cardiac risk as well as the clinical implications of ERP in ED patients.

## CONFLICTS OF INTEREST

T.C.F, M.K.C, and L.C have no conflicts of interest to declare. H.K.J is supported by a grant from the Novo Nordisk Foundation, Denmark (NNF18OC0031258) and received lecture fees from Abbott, Denmark, and Biosense Webster, Europe.

## AUTHOR CONTRIBUTION

TCF contributed to data collection, data management and analyses, statistical analyses, and writing of the manuscript. MKC contributed to data analyses, supervision, and revision of the manuscript. LC contributed to data collection, supervision, and revision of the manuscript. HKJ contributed to supervision and revision of the manuscript.

## Data Availability

Research data are not shared.
